# PARADIM: A Platform to Support Research at the Interface of Data Science and Medical Imaging

**DOI:** 10.1007/s10278-025-01554-y

**Published:** 2025-06-03

**Authors:** Yannick Lemaréchal, Gabriel Couture, François Pelletier, Ronan Lefol, Pierre-Luc Asselin, Samuel Ouellet, Jérémie Bernard, Leyla Ebrahimpour, Venkata S. K. Manem, Johanna Topalis, Balthasar Schachtner, Sébastien Jodogne, Philippe Joubert, Katharina Jeblick, Michael Ingrisch, Philippe Després

**Affiliations:** 1 Département de physique, de génie physique et d’optique, Université Laval, Québec, Québec Canada; 2 Centre de recherche de l’Institut universitaire de cardiologie et de pneumologie de Québec-Université Laval, Québec, Québec Canada; 3 Centre de recherche du CHU de Québec-Université Laval, Québec, Québec Canada; 4 Department of Medical Physics, Memorial Sloan Kettering Cancer Center, New York, NY USA; 5 Department of Radiology, LMU University Hospital, LMU Munich, Munich, Germany; 6 Munich Center for Machine Learning (MCML), Munich, Germany; 7 Institute of Information and Communication Technologies, Electronics and Applied Mathematics (ICTEAM), UCLouvain, Louvain-la-Neuve, Belgium

**Keywords:** PACS, DICOM, FAIR principles, Research data management, Digital infrastructure

## Abstract

This paper describes PARADIM, a digital infrastructure designed to support research at the interface of data science and medical imaging, with a focus on Research Data Management best practices. The platform is built from open-source components and rooted in the FAIR principles through strict compliance with the DICOM standard. It addresses key needs in data curation, governance, privacy, and scalable resource management. Supporting every stage of the data science discovery cycle, the platform offers robust functionalities for user identity and access management, data de-identification, storage, annotation, as well as model training and evaluation. Rich metadata are generated all along the research lifecycle to ensure the traceability and reproducibility of results. PARADIM hosts several medical image collections and allows the automation of large-scale, computationally intensive pipelines (e.g., automatic segmentation, dose calculations, AI model evaluation). The platform fills a gap at the interface of data science and medical imaging, where digital infrastructures are key in the development, evaluation, and deployment of innovative solutions in the real world.

## Introduction

Significant public and private investments have been made in recent years to advance artificial intelligence (AI), a technology that is now considered disruptive in several fields. In the medical domain, the potential of AI is especially high in image-intensive sectors such as radiology, nuclear medicine, radiation oncology, and pathology, building on impressive achievements at the intersection of deep learning and computer vision [2]. The development of AI models has been the primary focus of most efforts so far, in academia and industry. Meanwhile, foundational elements that support the development, evaluation, and deployment of AI, for instance, data and digital infrastructures, have received less attention and far fewer resources. Digital infrastructures include hardware, but also—and importantly—the software layers required to manage data, to automate computing tasks, and to address cybersecurity challenges, among others. Hardware arguably received the lion’s share of investments, leaving scarce resources for initiatives in Research Data Management (RDM) and research software/platforms supporting AI-related activities outside of model development.

Currently, AI models in the medical domain are often trained and evaluated using either a limited number of public datasets or in-house hospital datasets. Public datasets frequently suffer from issues such as a lack of transparency and insufficient quality assurance in data selection, preparation, and labeling (see, e.g., [3]). In-house datasets, on the other hand, are typically inaccessible for broader validation and reuse. These limitations contributed to the development of numerous machine learning models that struggled to generalize to real-world medical data (see, e.g., [4]). Shady datasets of unknown origins certainly contributed to the reproducibility crisis in AI, undermining trust in this technology (see, for instance, the concept of Frankenstein datasets [5]). Nonetheless, AI models are becoming increasingly mature—and a commodity in some respect that can be fetched from specialized outlets (e.g., Hugging Face [6], MONAI [7]) and fine-tuned for specific purposes. What is often lacking is high-quality data to train them properly, as underlined by advocates of the data-centric AI movement [8].

For AI to fully realize its potential in healthcare, it is essential to design and use digital infrastructures that enforce data quality and support privacy-preserving operations on these data while adhering to best practices in data (and model) management. This endeavor is best embodied by the MLOps culture (continuous integration of machine learning and operations, itself derived from DevOps) [9], where procedures related to the development and deployment of AI applications are unified and automated as much as possible, and by the related DataOps concept (continuous integration of data-related activities and operations) [10].

Several platforms have already been developed to support research in medical imaging, some of them available through free and open-source licenses. The ChRIS Project, for instance, proposes a framework to develop imaging plugins that is heavily oriented towards containerization and cloud computing [11]. XNAT is another platform, initially designed for neuroimaging, that offers several functionalities for research at the interface of data science and imaging [12]. XNAT uses the Open Health Imaging Foundation (OHIF) viewer [13], notably to facilitate quantitative imaging studies [14]. The Canadian Brain Imaging Research Platform (CBRAIN) [15] offers image-related functionalities, mostly geared towards the neuroscience community. The AWESOMME [16], Studierfenster [17], and CIRCUS [18] are other examples of platforms dedicated to the analysis of medical images. The OsiriX Foundation [19] also developed software to support research in medical imaging, notably to de-identify (Karnak [20]) and manage image collections (Kheops [21]). Finally, commercial cloud providers such as Google [22] and Microsoft [23] also offer medical imaging data hosting and associated analysis services.

Although numerous solutions exist that could fulfill most business needs (e.g., XNAT, ChRIS, CBRAIN, or AWESOMME), few offer a comprehensive suite of functionalities in a unified yet modular platform. Many platforms use non-DICOM image formats such as MINC, NIfTI, Analyse, or BIDS, relying on converters that potentially destroy the original and valuable metadata natively contained in DICOM objects (e.g., dates, acquisition parameters, and operators). Existing platforms typically treat images as files living in a directory, as opposed to objects that can be queried from a dedicated information system such as a Picture Archiving and Communication System (PACS) that is normally used in a production environment to manage medical images. Drifting away from the DICOM and PACS ecosystem is a risky business that could lead to identity errors, poor traceability, and inefficient data organization in general. The seemingly simple task of contouring anatomical structures in medical images, for example, can quickly lead to situations where the important information on who did the annotation is lost (e.g., intern, senior radiologist, or algorithm). Losing track of this type of metadata can heavily impact downstream analysis, for instance, supervised learning, where high- and low-quality data cannot be distinguished. Strict compliance with DICOM is also aligned with the MLOps approach as results from models packaged in this format can seamlessly be pushed back to the source PACS and made rapidly available to end-users through regular clinical systems.

This paper presents PARADIM (Platform for the Annotation, Reuse, and Analysis of Medical Images, or Plateforme d’Annotation, de Réutilisation et d’Analyse D’Images Médicales, in French) [24], a digital infrastructure that, from a high-level perspective, responsibly exposes high-quality medical image collections to users and facilitates streamlined and automated execution of large-scale operations on these collections (https://paradim.science). PARADIM, developed since 2019 [25, 26], is designed to address the needs of the medical imaging research community by (1) ingesting and de-identifying images according to specific profiles; (2) grouping images into collections; (3) creating contextualized annotations on images (why, who, when, and how); (4) controlling and monitor access to image collections; (5) securely exposing data to authorized users and machines; (6) orchestrating operations on images.

PARADIM was built with a strong emphasis on research data management best practices and strictly complies with the DICOM standard to ensure interoperability and reusability. It relies on resilient technologies for storage and deployment, can streamline data curation activities such as patient de-identification or image annotations, and execute traceable and automated data processing pipelines (e.g., AI-based segmentation, radiomic analysis, or dose calculations). Since the research community using PARADIM includes individuals with limited technical backgrounds, some functionalities were implemented with a strong focus on user-friendliness. From a governance perspective, PARADIM implements a data management framework that defines roles (e.g., stewards, curators, and managers) and associated responsibilities, as well as cybersecurity policies such as multifactorial authentication.

To summarize, PARADIM fulfills the following research needs (which are similar to those enumerated by Doran et al. [14] in the context of the XNAT platform):

- Images must be managed and curated as collections assembled under specific approvals (e.g., by Research Ethics Board), by a nominated data steward;
- Access to images must be monitored and managed through authentication and authorization mechanisms;
- Images must be de-identified before leaving the clinical realm, in ways that often depend on the project [27] (a one-size-fits-all approach is not possible);
- Relevant clinical or research data should ideally be stored along with images inside DICOM objects to minimize the risk of errors;
- Results derived from images should be packaged in standard DICOM objects along with their associated metadata (e.g., software versions).

This paper presents the principles that guided the design of PARADIM, a description of core functionalities, three use cases, and finally a roadmap to address current shortcomings. Major contributions include a description of research data management best practices in the context of medical imaging and technical details on a system architecture allowing modular development and evolution of the platform.

## Methods

PARADIM was designed with a best-of-breed approach, where components were primarily chosen for their DICOM compatibility and functionalities. Components were assembled to make PARADIM modular and API-based, allowing us to potentially replace parts without affecting the rest of the platform. Strict DICOM compliance along the entire chain of operations is a distinctive aspect of PARADIM.

### Guiding Principles

### FAIR Principles

The development of PARADIM was inspired and guided by high-level principles, first and foremost the FAIR (Findable, Accessible, Interoperable, Reusable) data management principles [28]. Funding agencies and international consortia are increasingly aware of the importance of best practices in RDM and adopted policies to promote them. These policies, often inspired by open science [29], are in some cases referring explicitly to the FAIR principles and are now mandatory in some funding programs (see, e.g., [30]). The fundamental driver of FAIRness in PARADIM lies in its interoperability component, embodied in its strict compliance to the DICOM standard for the entire data lifecycle. DICOM enables the seamless exchange and communication of information across different devices and software platforms from various vendors. Since DICOM is an open, well-documented standard, it satisfies FAIR’s accessibility requirements. Findability is fostered by the persistent IDs used by the DICOM standard (e.g., the Study Instance UID (0020,000D) tag that uniquely identifies an imaging study) and through the addition of schema.org markup (JSON-LD) in the PARADIM webpage, making Dataset objects discoverable by search engine crawlers. Finally, the reusability of data is at the core of PARADIM, which hosts carefully curated data collections that can be used to train or evaluate AI models, if appropriate approvals were granted. Reusability is fostered by documenting the data as much as possible by capturing extensive metadata throughout the entire chain, from contextualized annotations (who, when, how) to well-documented results. For instance, results of analyses are typically packaged into DICOM objects, along with details (e.g., model version) stamped in appropriate tags (e.g., the Software Versions (0018,1020) tag).

**Fig. 1:**
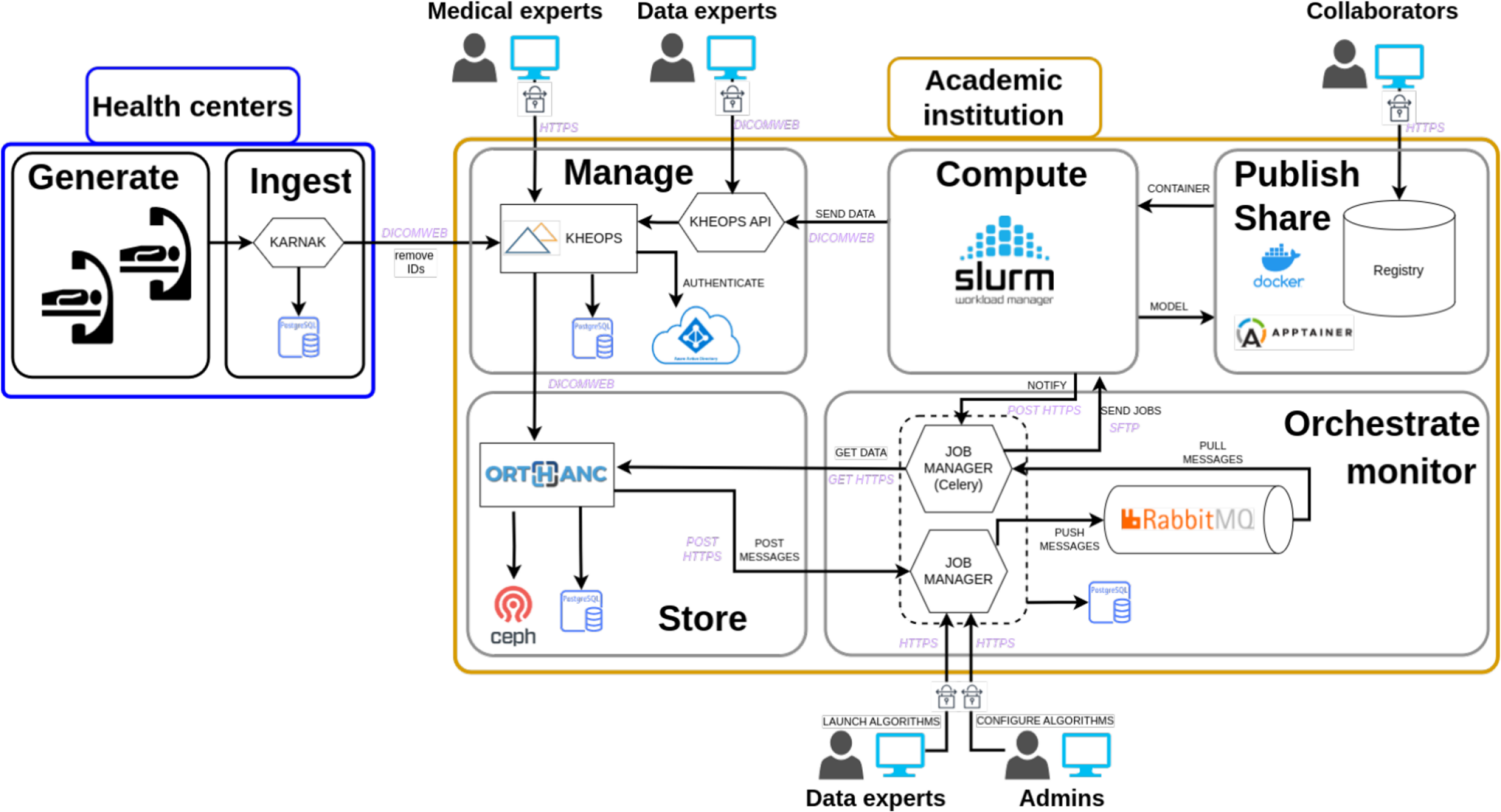
High-level system architecture of PARADIM. The de-identification component (Karnak) lives in the clinical domain (blue zone), while the rest of the platform (orange zone) is hosted in an academic environment

### Privacy by Design

Pooling large amounts of sensitive health data is not without risks. Research participants, funding bodies, Research Ethics Boards (REB), citizens at large, and other stakeholders all expect personal health information (PHI) to be managed and used responsibly, in compliance with laws and best practices. Within PARADIM, data is de-identified upon ingestion (see Section “Data Ingestion and De-Identification”) following recipes prescribed by the DICOM standard. Even de-identified, images are still considered sensitive data and access policies are enforced. A REB-approved data management framework clearly defines the roles and responsibilities of all stakeholders and describes the possible uses and conditions for access (e.g., a collection can only be used for an REB-approved project; if other uses are desired, they must be approved separately by the REB). Image collections are only accessible to users who have received explicit authorization. Each collection is managed by a data steward who controls user rights (e.g., to add or remove data). By default, new users have no access to collections. In PARADIM, identities are managed through our institutional Microsoft Entra ID instance, providing single sign-on (SSO) and multifactor authentication (MFA) functionalities. Authorized users are listed in a specific security group. Revocable access tokens can be generated by data stewards to allow external agents (machines) to access specific data collections through a REST API.

### Free and Open-Source

PARADIM was mostly built from free and open-source components, with the exception of the institutional authentication service (Microsoft Entra ID) provided by a commercial vendor and used for convenience but that could easily be replaced by an open-source solution such as Keycloak [31]. The platform is primarily composed of pre-existing components and is complemented by locally developed components. Assembling the platform from open-source components allowed for agility and flexibility in the development process. Modularity is central in the platform’s architecture, with APIs linking components together. While the platform is currently deployed in RedHat OpenShift [32], which is a vendor-specific environment, it maintains standard compatibility with Kubernetes and could easily be ported to other environments through a lift and shift operation. Avoiding vendor lock-in was a guiding principle throughout the development process.

### MLOps

The development of PARADIM was guided by the MLOps/DataOps approach [9, 10], which translates in our context by integrating research activities as much as possible into healthcare operations. PARADIM was designed to be seamlessly connected to the clinical environment. This facilitates the ingestion of real-world data (e.g., from PACS) and the exposition of AI models and other research products to end-users through the existing clinical IT infrastructure. The platform supports knowledge transfer activities in AI, as models can easily be inserted in pipelines feeding clinical activities (e.g., push back DICOM objects to the clinical PACS).

**Fig. 2:**
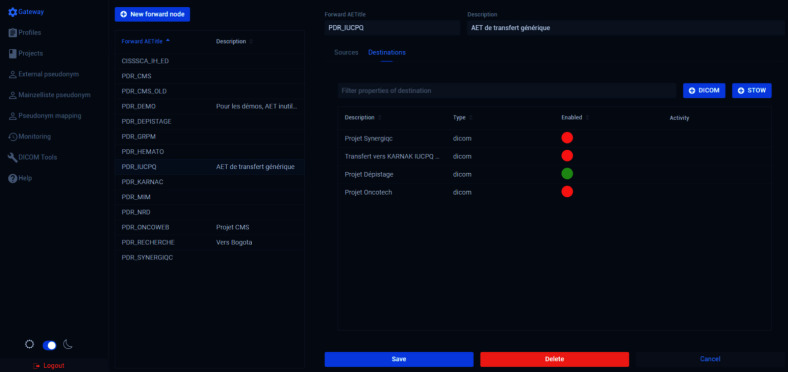
The Karnak web interface, allowing the management of de-identification profiles and the routing of incoming images to specific collections

### Overview of the Platform

The PARADIM architecture is presented in Fig. 1, showing the main technological components of the platform as well as high-level functionalities (ingest, manage, store, compute, orchestrate/monitor, and publish/share). Components are deployed in two distinct environments (blue—health centers and orange—academic institution in the figure), corresponding to the clinical and academic network areas, respectively. Purple text corresponds to the communication protocols between components, which all use an encryption layer. Access to the platform is restricted to IP addresses belonging to our academic institution (Université Laval), including through VPN services.

### Data Ingestion and De-Identification

In many jurisdictions, including ours, data containing PHI can only reside in clinical network area. They must be de-identified before they can be transferred outside this domain. The de-identification component of PARADIM (Karnak) therefore lives in the blue zone in Fig. 1. The Karnak software [20] was developed by the Osirix Foundation and is used in PARADIM as a DICOM de-identification gateway. It is the only component in PARADIM located within the clinical domain (blue zone—health centers in Fig. 1) and handling PHI. Karnak provides several functionalities related to data ingestion and de-identification, which is not a trivial task considering the large number of DICOM tags that can contain PHI. Although the basic de-identification recipe provided by the DICOM standard (PS3.15) is adequate for most cases, some projects might need information that would otherwise be deleted or altered by this process [27]. Within Karnak, de-identification profiles can be defined to meet the requirements of each project. These profiles prescribe which DICOM tags must either be removed, shifted by introducing a random time offset, replaced by a constant value, or encoded (e.g., using hash-and-salt). The default profile in Karnak is the Basic Application Level Confidentiality Profile from the DICOM standard.

Additional tags can also be added to profiles to follow the FAIR principles and document data as much as possible. This Karnak feature is used, for instance, to stamp project information in images, such as the consented use in the Distribution Type (0012,0084) tags, whose possible values are NAMED_PROTOCOL, RESTRICTED_REUSE, and PUBLIC_RELEASE. The de-identification profile used itself is recorded in the De-identification Method Attribute (0012,0063) tag. Project information (including the REB approval number) is documented in dedicated DICOM tags of the Clinical Trial Study DICOM module. This marking of images upon ingestion facilitates long-term management, notably with respect to REB or legal approvals and conservation times.

Karnak is capable of generating new IDs for patients using salted hashes or use a pre-defined mapping provided by data stewards. It can also hide PHI that was burned in images by using black pixels within a rectangular zone of a specific size and position.

**Fig. 3:**
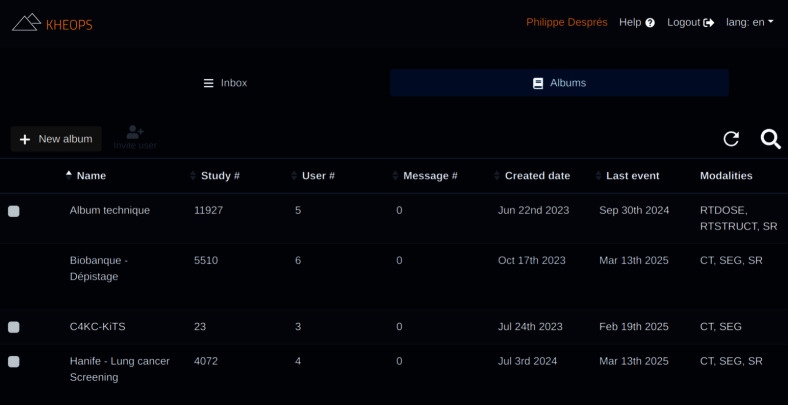
The Kheops data management web interface, showing collections (albums) the user has access to, along with content information

Karnak acts as a DICOM listener. Modalities can be configured to push studies directly to this destination, or a third party might orchestrate the C-FIND and C-MOVE operations for image retrieval. The latter is most commonly used, where a PACS/DICOM server is used to find specific studies and initiate the transfers. We use scripts and the Orthanc DICOM server [33] as a Swiss army knife to perform these operations. Typically, users provide a list of patient IDs, record numbers, dates, or modalities of imaging studies they want to retrieve, and scripts are used to query the PACS and initiate transfers.

In addition to de-identification, Karnak is used to route incoming images to the next stage, namely storage within a collection (cf. next section). This routing is based on filters applied to selected DICOM tags. For example, an incoming chest CT study from the ER for patients over a given age might be routed to a specific image collection. Figure 2 shows the Karnak web interface.

### Data Management

De-identified data are transferred from Karnak to the main PARADIM ecosystem (orange zone in Fig. 1), where they are received by the Kheops component [21], also developed by the Osirix Foundation. In Kheops, images are managed in so-called albums (collections of DICOM studies and series), which boils down to database tables (PostgreSQL) where images and collections are linked by a one-to-many relation. Kheops also keeps metadata about collections in its dedicated database. Images stored in PARADIM can belong to multiple collections, which avoids the duplication of DICOM objects. Collections typically pool images related to research project cohorts, to specific pathologies, or to types of examinations. For example, PARADIM hosts separate collections for two screening programs in Québec, Canada: low-dose CT images for lung cancer and mammograms for breast cancer. A data steward is designated for each collection and is the only person controlling access and permissions, including adding or deleting images. To prevent data loss, delete operations in Kheops only dissociate images from a collection. Images are not deleted on the storage backend (cf. Section “Data Storage”), unless the data steward or another authority body instructs the destruction of images.

Kheops is not used as the storage backend itself, but acts as an authentication and authorization layer that manages access to data with collection-specific access rights, both from a web interface and a REST API based on the DICOMweb protocol. It is the entry point to the platform, both for humans and software access. Humans authenticate through an institutional single sign-on (SSO) service (Microsoft Entra ID), with multifactorial authentication enforced. Kheops also allows data stewards to generate revocable access tokens so that machines can access images. The Kheops web interface is shown in Fig. 3.

**Fig. 4:**
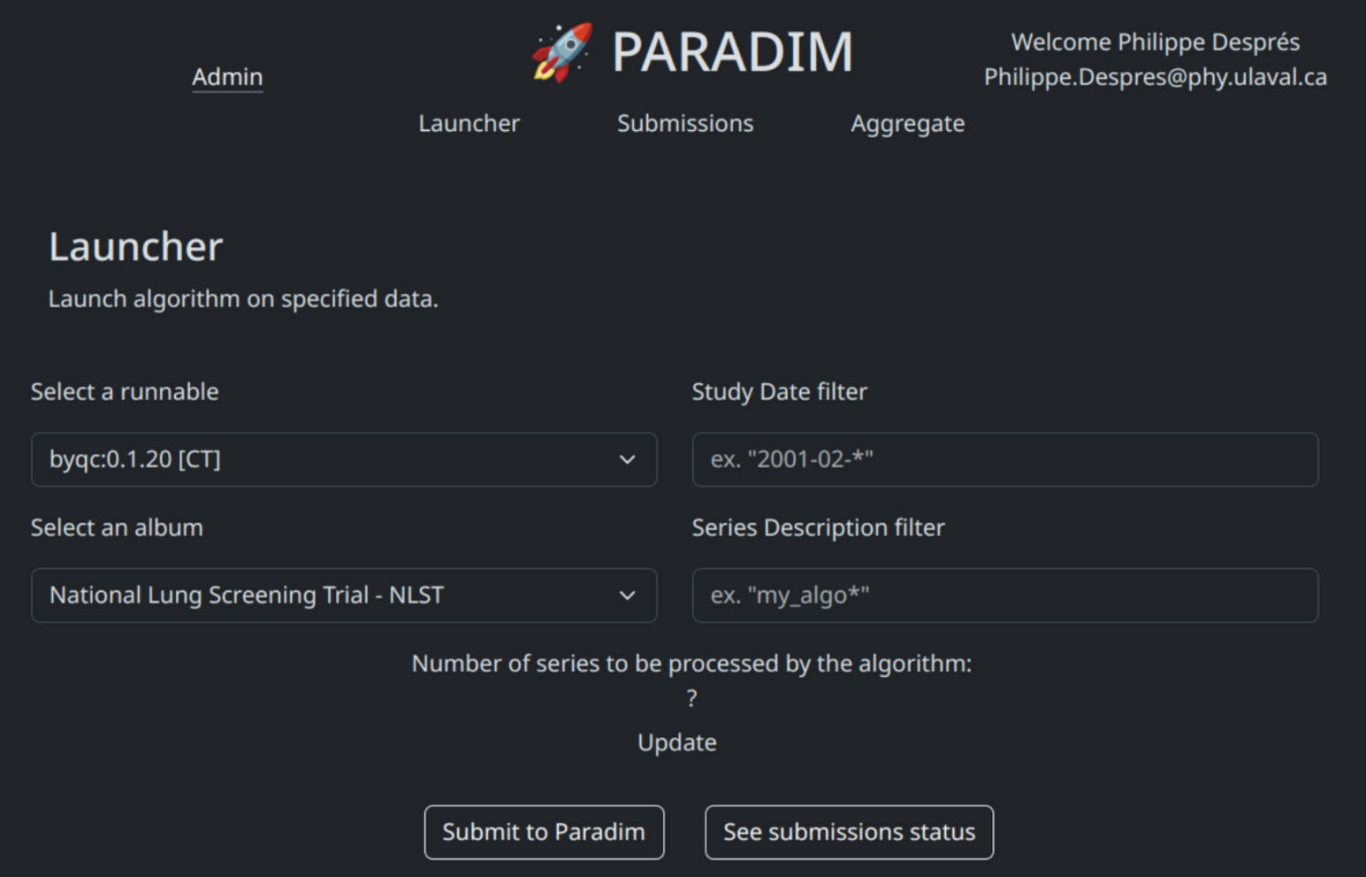
Interface of the Job-Manager component of PARADIM, where users can manually launch jobs on collections or subpart of it

### Data Storage

The backend storage of PARADIM is Orthanc, a free and open-source DICOM ecosystem [33]. Orthanc is well-suited to serve as a DICOM data store but has limited functionalities for data governance, including user management, authentication, and authorization. In PARADIM, these functionalities are handled by Kheops, as explained above. Orthanc was historically the core of the PARADIM platform [26], upon which additional functionalities were built.

The plugin architecture of Orthanc is well-suited for the modular approach adopted for PARADIM, as it can be customized and extended without modify the product core. Orthanc plugins configured in PARADIM are

- Python plugin [34], to launch custom scripts that will notify the arrival of new studies and series and to add new endpoints to the existing API;
- DICOMweb plugin, to provide a unified interface for communication between Orthanc and Kheops, since Orthanc is not exposed to users;
- PostgreSQL plugin, to use PostgreSQL database as a storage backend for configuration and metadata;
- AWS S3 plugin, to use Ceph storage (S3 compatible object storage from RedHat) provided by our institutional IT services.

Orthanc stores the main DICOM tags of images in a database for rapid filtering and retrieval operations. Additional DICOM tags can be indexed in the database with a simple modification to the configuration file of Orthanc (cf. option ExtraMainDicomTags). In the context of PARADIM, the indexation of additional DICOM tags was used to optimize the interfacing with external components, notably the OHIF image viewer [13]. Because DICOM instances are immutable, additional information, such as annotations, segmentations, or derived information, is stored as distinct DICOM objects within the same study in Orthanc (physically in the S3 object store).

### Job Orchestration

Given the increasingly crowded landscape of AI models, it is important to rely on robust, traceable, and automated methods to generate results (e.g., inference, training). One of the added values of PARADIM lies in its ability to automate processes and jobs, which is aligned with the MLOps/DataOps philosophy. Orchestration components were developed in-house, with these requirements in mind. They provide functionalities to automatically trigger computation jobs and to capture execution artifacts (e.g., logs) and metadata such as software and model versions (using the DICOM Software Versions tag). Execution logs, for instance, are kept as DICOM-SR objects (structured reports) and stored within the same study as the original DICOM instances for traceability.

A component named Job-Manager was developed in-house using the Django Python framework to allow users to launch jobs interactively through a web interface or programmatically through a REST API [35]. In the background, Celery [36] is used to manage these jobs. In typical scenarios, Orthanc sends a contextual message (e.g., a new lung CT study was received) to the Job-Manager API upon receiving new images. The message contains the Series Instance UID (0020,000E) tag, the Study Instance UID (0020,000D) tag, and information about the source modality. For some modalities, jobs are systematically dispatched. For example, an incoming CT series always triggers a segmentation job, currently using the TotalSegmentator model [37]. All the code used to perform actions on DICOM objects is packaged as containers stored in a private Docker registry, with a naming convention using task name and semantic versioning (e.g., total_segmentator:0.1.22). These containers must comply with the Open Container Initiative (OCI) specifications. Internally, the platform converts these user-provided containerized tasks into Apptainer images (formerly known as Singularity) for execution.

**Fig. 5:**
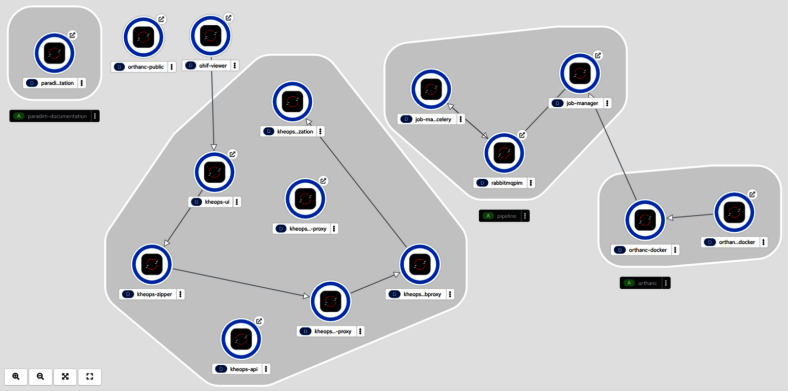
Topology of the deployment within our institutional Kubernetes environment (RedHat OpenShift)

The Job-Manager component generates a JSON object from the Orthanc message and the requested task/version pair, which is then sent to a RabbitMQ message bus [38] acting as a buffer that prevents the overloading of computational resources. Messages accumulated in the bus are consumed one by one by the Job-Manager (Celery) pods, which also verify if results for a specific task/version pair are already cached in a technical collection in Kheops gathering all results generated. If so, the results are added to the target collection instead of being regenerated. Otherwise, the Job-Manager will prepare a compute job to be queued by SLURM on our academic HPC cluster along with corresponding data. The job preparation process consists of (1) retrieving DICOM files, (2) generating a SLURM script with appropriate parameters (e.g., CPUs, memory, or queue policy), and (3) sending it to the HPC cluster.

It is also possible to trigger a specific task/version pair manually from the web interface (Fig. 4). Authenticated users choose the task/version pair to run on a collection (or a part of it) they have authorization to read. The message sent to the Job-Manager is the same as the Orthanc message, plus the target collection identifier. If results for a particular task/version pair already exist, they will not be regenerated. Existing ones will be added to the target collection.

On PARADIM, all results are packaged as DICOM objects to be pushed back to a collection and natively managed by the DICOM server. This is relatively straightforward for some results with dedicated DICOM specifications, such as DICOM-SEG or DICOM RT-STRUCT for segmentation. Otherwise, users are encouraged to package their results in DICOM-SR objects, which can be used to embed any type of information (sample source code is provided to this end [39], along with other snippets and utility functions in the PyOrthanc package [40]).

### Deployment Environment

The deployment of PARADIM is versioned, scheduled, and managed using Gitlab [41] CI/CD methods. Current deployment occurs in an institutional OpenShift (RedHat) Kubernetes environment, except for the PostgreSQL databases and Ceph storage, which are managed by institutional IT, and Karnak (de-identification), which typically lives on a virtual machine in the clinical domain. OpenShift provides a web-based interface and REST API functionalities used for the orchestration, administration, and monitoring of PARADIM (Fig. 5). Files in YAML format define the different Kubernetes resources to deploy and the computational requirements. It also includes the number of replicas to deploy to ensure high availability, as well as the network configuration, such as the TCP/IP ports to open.

All applications can be scaled, except for the RabbitMQ component, to avoid concurrent access problems. Resources can be quickly redeployed or terminated using the GitLab task scheduler. Probes were implemented to periodically query the application status. In case of failure, notifications are sent by email using the SysDig monitoring tool provided by our institution. Sensitive variables and information, such as database access, are managed with the GitLab Secrets Manager and dynamically inserted into Kubernetes secret files during deployment. Deployed instances, called pods in the Kubernetes nomenclature, are accessible within the platform through services, and from the external network through Openshift routes. For example, Orthanc has no external route, and Kheops accesses it through a service. However, Kheops is open to the external network (and users) through routes. The platform lives inside the institutional network, accessible either from campus or through a VPN connection. In all cases, connections are made with the TLS security protocol with institutional certificates.

There are currently two PARADIM deployments: one for a stable production environment and another dedicated to experiments and explorations. As stated in Section “Guiding Principles,” advanced OpenShift functionalities were not used in order to keep everything vanilla from a Kubernetes perspective and facilitate migration to other service providers.

## Results

This section describes three use cases that demonstrate some functionalities of PARADIM, which has mostly been used so far as a data curation and model inference platform.

### Data Annotation and Segmentation

Annotated datasets remain central in AI model training and evaluation in medical imaging. The process of gathering annotations can be considered expensive in terms of time and expertise, and it is crucial to preserve such annotations in the long term by implementing best practices for data management and curation. According to FAIR principles, it is essential to document as much as possible the context of the data capture process (e.g., who, when, how). For image annotations, which often come in the form of segmentations and contours delineated by specialists, producing such documentation is critical to preserve information that could hint at data quality (e.g., senior radiologist vs student intern) and to keeping track of how the data was generated (e.g., which software or clinical guideline was used). This is even more true now as algorithms are increasingly used to generate annotations and labels (e.g., TotalSegmentator); keeping track of *human* ground truths appears crucially important.

Minimal requirements for annotation metadata include the name of the annotator (which can be stored in the dedicated Content Creator Name DICOM tag) or the software/version pair used for AI models that autonomously produce the content. Tools fulfilling these requirements (combining annotation functionalities and strict DICOM compliance) are relatively rare, with OHIF [13] and ePAD [42] being potential candidates. For PARADIM, we opted for the 3D Slicer [43] software and its QuantitativeReporting plugin [44]. 3D Slicer is used to fetch data from Kheops (through authenticated DICOMweb transfers), to capture the name of the operator (QuantitativeReporting plugin functionality), to perform lesion contouring tasks, to encode everything in DICOM RT-STRUCT or DICOM-SEG objects, and to push these results back into Kheops. The original QuantitativeReporting plugin [45] was forked to allow the naming of anatomical structures or lesions in a configuration list. A fork [46] of RT-STRUCT manipulation library [47] was also used in the creation of the annotations. The annotation pipeline in PARADIM is routinely used in our institution to create segmentations on images that serve as ground truth for model training or evaluation.

### Automated Processing Pipelines

PARADIM facilitates the construction of automated processing pipelines, which are automatically triggered when new imaging series arrive. As a proof of concept, the TotalSegmentator model [37], capable of segmenting major anatomical structures in CT and MR images, was integrated in a pipeline that is triggered each time a CT series of the thorax is received by the platform. The Orthanc component, through its Python plugin, triggers the Job-Manager component that prepares the data and the computation task. This pipeline automatically generates new DICOM RT-STRUCT objects in the study, along with relevant metadata (e.g., execution log and software version). The Job-Manager database keeps track of objects created by a particular task/version pair so that subsequent calls will not invoke a new computation. This MLOps/DataOps approach allows for large-scale computational endeavors and continuous model performance monitoring.

The Job-Manager also provides an interface to manually trigger jobs on a particular series or collection. This was used, for instance, in the evaluation of AI models for the task of detecting pulmonary nodules in low-dose CT images. The scores associated with each AI-detected nodules were reported in DICOM-SR objects using the Content Sequence (0040,A730) tag. The DICOM-SR objects are then stored inside their originating study on the platform. It is relatively straightforward afterwards to implement result aggregation functions on PARADIM; users package their code in a container, which can thereafter be applied to a collection (e.g., to generate statistics). In the case of the AI-based nodule detection study, models were evaluated by comparing their predictions to reference standard segmentations readily available on the platform. All operations and data containers remain DICOM-based during the entire process. PARADIM was also used similarly to support the analysis of radiomic features extracted from CT images [48].

### Monte Carlo Simulations

While PARADIM was built with AI model development and evaluation in mind, other use cases can also benefit from the infrastructure. One such case is a Monte Carlo dose calculation pipeline in brachytherapy, where DICOM CT images, treatment planning specifications (as DICOM RT-PLAN instances), and segmentations (as DICOM RT-STRUCT instances) are used to generate dose maps (as DICOM RT-DOSE instances) and dosimetric indices. The user code provided as a container was made available to the Job-Manager through its registry. Each incoming DICOM RT-PLAN on PARADIM triggers the execution of this calculation pipeline, which is extensively described in a dedicated publication [49]. This kind of automation streamlines the production of results and fosters the transition from research to the real world, through robustness and reproducibility. In this particular case, it allows the systematic production of new, potentially more accurate dose maps alongside those routinely used in the clinic. From a user perspective, the complexity related to storage and computing resource dispatching was hidden by PARADIM so that efforts could be focused on data analysis instead of data management.

## Discussion and Future Directions

The implementation of PARADIM was inspired and guided by the FAIR principles, by privacy and security best practices, by the free and open-source philosophy, and by data-centric AI. It aims to instill the MLOps/DataOps to the field of medical imaging, building upon the widely used DICOM standard. Best practices for data management are increasingly needed in the data-driven research landscape to avoid situations where, for example, poor data traceability leads to questionable clinical or research results. For the particular case of contouring lesions on images, capturing the annotator’s identity led us to unveil discrepancies in methods used by different individuals, which is an important information that can impact the generated results. Considering that machines are poised to increasingly perform this type of task in the future, keeping the record of the origin of a piece of information is of paramount importance, if only for distinguishing human-produced ground truths from other annotations.

PARADIM is still under development as new needs arise and new software components emerge that could advantageously replace the current ones. The modular and API-based design of PARADIM was purposely chosen to facilitate the future evolution of the platform, where components could be replaced with minimal impact on the others. The current annotation procedure, for instance, is not ideal, and work is being conducted to replace it. The use of a desktop-based client (3D Slicer) has some advantages, including advanced segmentation utilities, but remains at odds with the rest of the web-based, API-driven components. Even if secure, dedicated workstations are used to run 3D Slicer, this software necessitates data to be locally downloaded, while they should ideally remain within the realm of PARADIM at all times. Ongoing work aims at designing a web-based contouring platform allowing the generation of DICOM objects along with relevant metadata, including the annotator’s identity.

The Karnak component used for de-identification turned out to be very convenient, allowing filter-based image routing, fine control over tags containing PHI, and the embedding of additional metadata upon ingestion (e.g., REB approval number related to the project). Moreover, Karnak can generate new IDs based on a salted hash, ensuring that the same ID string is generated for a given patient ID. Some collection owners, however, have their own lookup table to translate a patient ID (typically an institutional Medical Record Number) to a de-identified one. Karnak can handle these cases but do not persist the lookup table, leading to manual operations in some cases. Work is being done to refactor Karnak and improve some functionalities, including those related to identity management.

Kheops also offers several convenient functionalities to support data management. Its interface provides a user-friendly experience for clinicians and users with limited technical expertise. Data governance is easy to implement within Kheops, and non-technical data stewards can grant access and privileges to specific users within a collection. With the concept of collections in Kheops, it is also straightforward to implement worklists where annotators do not have access to the content generated by each other. Kheops, however, is relatively complex under the hood, with six microservices handling the queries and the data. This complexity sometimes leads to glitches, including timeouts that allowed series with missing instances (images) to be imported without warning. The number of services involved makes Kheops relatively difficult to troubleshoot. We have implemented a lightweight web application that re-implements the DICOMweb STOW-RS route to make the image ingestion process more robust. This route uses the same authentication tokens as our Kheops instance, making either route equivalent. For heavier operations, we prefer to interact with Orthanc directly using Python scripts with the PyOrthanc library [40]. With the recent introduction of the concept of labels in Orthanc, we hope to reproduce the Kheops functionalities natively within the Orthanc DICOM server, which remains at the core of the platform.

Although the PARADIM platform was developed to be deployed in any Kubernetes environment, this has not yet been tested extensively outside of OpenShift (RedHat). Deployment within a commercial cloud environment is currently ongoing.

PARADIM has been mostly used so far as an inference platform, but model training tasks are increasingly common, bringing additional challenges for the future.

## Conclusion

PARADIM fills a gap at the interface between AI (and other tasks) and medical imaging, where data and digital infrastructures were historically not given the attention they deserve. It was designed to store, manage, enrich, and responsibly access medical images in a data science context. It relies extensively on the DICOM standard for maximal interoperability and facilitates the orchestration of analysis pipelines. PARADIM is currently used by several research teams (currently 10 REB-approved projects) for various tasks (e.g., annotation, AI model training, AI model evaluation) and keeps track of as much information as possible on data operations, aligned in this regard with the FAIR principles of research data management. The platform offers a comprehensive suite of tools and services for data governance and curation, allowing researchers to focus on their projects without worrying about data management issues. PARADIM facilitates collaborations, allowing multiple parties to bring their analysis pipelines to the data and not the other way around, so that data stewards control the data at all times.

## Data Availability

No material involved in this work.

## Code Availability

References to code are provided as citations in the text.

## Data Availability

Data collections hosted on PARADIM can be accessed following formal agreements with data stewards.
